# Making an effort to feel positive: insecure attachment in infancy predicts the neural underpinnings of emotion regulation in adulthood

**DOI:** 10.1111/jcpp.12198

**Published:** 2014-01-08

**Authors:** Christina Moutsiana, Pasco Fearon, Lynne Murray, Peter Cooper, Ian Goodyer, Tom Johnstone, Sarah Halligan

**Affiliations:** 1 Division of Psychology & Language Sciences, University College LondonLondon, UK; 2 School of Psychology and CLS, University of ReadingReading, UK; 3 Department of Psychology, Stellenbosch UniversityStellenbosch, South Africa; 4 Department of Psychiatry, University of CambridgeCambridge, UK; 5 Department of Psychology, University of BathBath, UK

**Keywords:** Emotion regulation, fMRI, infant attachment, longitudinal

## Abstract

**Background:**

Animal research indicates that the neural substrates of emotion regulation may be persistently altered by early environmental exposures. If similar processes operate in human development then this is significant, as the capacity to regulate emotional states is fundamental to human adaptation.

**Methods:**

We utilised a 22-year longitudinal study to examine the influence of early infant attachment to the mother, a key marker of early experience, on neural regulation of emotional states in young adults. Infant attachment status was measured via objective assessment at 18-months, and the neural underpinnings of the active regulation of affect were studied using fMRI at age 22 years.

**Results:**

Infant attachment status at 18-months predicted neural responding during the regulation of positive affect 20-years later. Specifically, while attempting to up-regulate positive emotions, adults who had been insecurely versus securely attached as infants showed greater activation in prefrontal regions involved in cognitive control and reduced co-activation of nucleus accumbens with prefrontal cortex, consistent with relative inefficiency in the neural regulation of positive affect.

**Conclusions:**

Disturbances in the mother–infant relationship may persistently alter the neural circuitry of emotion regulation, with potential implications for adjustment in adulthood.

## Introduction

For almost a century, a fundamental assumption of developmental psychology has been that persistent changes in psychological function that occur as a consequence of early experience are underpinned by equally persistent alterations in the organisation of the underlying neural circuitry (Gunnar, 2003; Kolb et al., 2012). In particular, animal research indicates that disruptions in the provision of early care alter the prefronto-limbic circuitry, resulting in corresponding alterations in emotional and behavioural responding (Caldji, Diorio & Meaney, 2003; Meaney, 2001). In humans, direct evidence linking early experiences to adult neural functioning is extremely limited. However, retrospective accounts by adults of exposure to early abuse and neglect suggest that *structural* changes may result, particularly in prefrontal and limbic regions involved in self-regulation of emotional responding (Andersen & Teicher, 2008). There are few equivalent studies examining *functional* neural activity, but prolonged early institutional rearing has similarly been linked to altered patterns of activation in the amygdala and regions of the prefrontal cortex (PFC) that subserve emotional responding (Chugani et al., 2001; Tottenham et al., 2011). Such observations are critical, as the ability to modulate emotions in response to situational demands facilitates effective cognitive, behavioural and social engagement; and abnormalities in emotion regulatory skills are present in various forms of psychopathology (Gross, 2007). It remains to be established whether less extreme variations in the quality of early care can have lasting effects on human brain development, and whether their effects specifically impact on the neural underpinnings of emotion regulation. Here, in a prospective longitudinal study we examined the quality of the mother–infant attachment relationship as a potential influence on the neural systems supporting emotion regulation in adulthood.

Attachment theory represents perhaps the most influential psychological model for understanding the role of early experience in long-term social and emotional adjustment (Ainsworth, Blehar, Waters & Wall, 1978; Bowlby, 1979). The security of the attachment bond between an infant and their primary caregiver is considered to be a fundamental aspect of the early caregiving environment, forming the foundation for subsequent socio-emotional development. Secure attachments, relative to insecure ones, appear to confer a range of developmental advantages, such as better peer relationships (Schneider, Atkinson & Tardif, 2001), fewer behavioural problems (Fearon, Bakermans-Kranenburg, van IJzendoorn, Lapsley & Roisman, 2010) and lower rates of affective disorder (Murray et al., 2011). A key hypothesis advanced to explain the long-term connections between attachment and socio-emotional functioning is that secure attachment promotes the development of effective emotion regulation (Cassidy, 1994). According to this account, securely attached infants experience interactions with their primary caregivers that are broadly positive and responsive to the infant's emotional needs, facilitating the development of flexible and effective emotion regulation. In contrast, insecurely attached infants are subject to inconsistent or negative caregiver responses, particularly when stressed, and correspondingly develop rigid and/or ineffective ways of regulating emotion. Resultant differences in the capacity to self-regulate emotions are assumed to be underpinned by persistent alterations in the underlying neural systems (Bowlby, 1979).

To date, a single study has examined the neural correlates of infant attachment and identified concurrent associations between insecure attachment and reduced left frontal EEG activity in young infants, consistent with reduced approach/reward related responding (Dawson et al., 2001). Longer term associations between infant attachment and neural functioning have not been studied, but such investigation is timely, as recent decades have seen marked advances in our understanding of the neural underpinnings of emotion regulation. A network of neural systems supporting emotion regulation has been broadly established, involving dynamic interactions between ‘bottom-up’ affective appraisal systems, such as the amygdala and parts of the basal ganglia, and top down appraisal systems of the prefrontal cortex (PFC) and anterior cingulate cortex (ACC) (Ochsner & Gross, 2007; Wager, Davidson, Hughes, Lindquist & Ochsner, 2008). Specifically, the ‘top down’ regulation of emotion involves areas of the PFC which modulate activity in subcortical regions that are directly involved in generating affective responding, particularly the amygdala and nucleus accumbens (NAcc).

Importantly, the extent to which the neural circuitry of emotion regulation is functioning effectively has implications for wider adaptation. For example, systematic alterations in the neural systems subserving emotion regulation are indicated in depression, the primary symptoms of which are profound negative affect (dysphoria) and/or a marked absence of positive affect (anhedonia). In particular, major depressive disorder has been associated with a failure in the top-down, prefrontal regulation of amygdala activity during effortful attempts to down-regulate (i.e., actively decrease) negative affect (Johnstone, van Reekum, Urry, Kalin & Davidson, 2007). The regulation of positive affect has been less studied. However, investigation of attempts to actively increase *positive* affective experiences in depressed individuals identified reduced frontal striatal connectivity and an associated failure to sustain NAcc responding to positive stimuli, relative to a non-depressed comparison group (Heller et al., 2009).

### The current study

We investigated whether attachment security during infancy directly predicts functional activity in the neural systems subserving emotion regulation in young adults. Participants (*n* = 54) were recruited from a longitudinal cohort of the offspring of mothers with postnatal depression (PND) and non-depressed controls, studied from birth (Murray, 1992). Attachment status was assessed in infancy using the Strange Situation. Neural responding was examined at 22-years via an established fMRI paradigm which isolates emotion regulatory processes in two key domains: active attempts to up-regulate positive affect and to down-regulate negative affect (Jackson, Malmstadt, Larson & Davidson, 2000). We tested the general hypothesis that infant attachment insecurity versus security would predict alterations in the engagement of selected brain regions previously established to be modulated in active emotion regulation paradigms (Heller et al., 2009; Johnstone et al., 2007). Therefore, we examined BOLD response in pre-defined regions of the PFC (orbitofrontal cortex, cingulate gyrus, superior, middle and inferior frontal gyri) and in the amygdala during attempts to down-regulate emotional responses to negative stimuli, where inverse associations between PFC and amygdala activity are expected. In addition, we examined equivalent PFC and NAcc activation during the up-regulation of emotional responses to positive stimuli, and the extent of co-activation between the two. These regions of focus for analyses were established *a priori* based on existing evidence regarding the neural circuitry involved in emotion regulation in both clinical and non-clinical groups (Johnstone et al., 2007; Ochsner et al., 2004; Phan et al., 2005).

We expected that the insecurely attached group, relative to their securely attached counterparts, would show less effective neural regulation of emotion 20-years later, expressed as significant group differences in the BOLD response in regions of interest. Specifically, previous research has identified two patterns of activation that are indicative of a lack of PFC regulatory control of affect (Erk et al., 2010; Johnstone et al., 2007): *either* inappropriate engagement of prefrontal regulatory circuitry which is relatively ineffective in modulating subcortical activity in the amygdala/NAcc; *or* generally lower activation of PFC. Thus, we tested for overall differences in the pattern of PFC activation between secure and insecure attachment groups, and also for related group differences in the strength of association between PFC and amygdala/NAcc activation. We controlled for key potential confounds where appropriate, namely the presence/absence of maternal PND, offspring history of depressive disorder, current depressive symptoms and gender. Finally, we examined whether later maternal parenting behaviour (assessed via observation at 5 and 8 years) could explain any infancy attachment effects.

## Methods

The study was approved by NHS and institutional ethical review bodies. All participants provided informed consent prior to taking part in the research.

### Participants

Participants derived from a prospective longitudinal study of the development of children of postnatally depressed and well women (Murray, 1992). The sample was originally recruited at 2 months postpartum, through screening a community sample of primiparous mothers of healthy, full-term infants for PND. The Edinburgh Postnatal Depression Scale (EPDS) (Cox, Holden & Sagovsky, 1987) was administered at 6 weeks postpartum, and women scoring over 12 were interviewed; 61 women who met research diagnostic criteria for depressive disorder were identified, 58 of whom were recruited for the study and 42 non depressed mothers were also recruited via random selection. At 22-years of age, 45 (76%) offspring from the PND group and 39 (93%) from the comparison group were available for the study. Of these, 63 were able to complete MRI scanning (exclusions were due to diabetes, epilepsy, metal implants and inability to attend a session at the University); emotion regulation task data were excluded for nine participants, due to structural abnormalities (*n* = 3), excessive movement (*n* = 4), technical problems (*n* = 1) and non-compliance with the protocol (*n* = 1). The final sample comprised 24 females and 30 males, mean age 22 years.

### Measures

#### Infant attachment

At 18 months infant attachment was assessed using Ainsworth's Strange Situation Procedure, the gold standard observational measure (Ainsworth et al., 1978). Attachment patterns were categorised according to the infant's response to separation from and reunion with the mother in an unfamiliar environment as secure, or insecure (i.e. avoidant, ambivalent or disorganised). Two trained raters coded videotaped assessments using standard procedures (Ainsworth et al., 1978; Main & Solomon, 1990). Cohen's kappa coefficient for the four possible attachment classifications of the two researchers who independently scored 63 randomly selected videotapes of the Strange Situation was .94. Any discrepancies were resolved by consensus agreement. Following the majority of previous studies, we based analyses on comparisons of secure versus insecure infants.

#### Maternal parenting at 5 and 8 years

At 5 and 8 years mother–child interactions were video-recorded in age appropriate contexts presenting a degree of challenge to the child. At 5 years, the interaction comprised a 10-min ‘snack’, where the child needed help with difficult to manage refreshments; at 8 years it comprised a 20-min maths-problem task. Interactions were scored by trained raters, blind to group, using age appropriate schemes to measure maternal emotional support, including ratings of warmth, acceptance, sensitive responsiveness and availability (Murray et al., 2011). Interrater reliability for these assessments was established (5 years intraclass *r *=* *.94; 8 years Cohen's *κ *= .94, each based on double coding of 10 randomly selected recordings); previous research with the same sample found lower maternal support when children had been insecurely attached as infants (Murray et al., 2011).

#### Depression

Depressive disorder was indexed via the Structural Clinical Interview for DSM-IV at 22 years (Spitzer, Williams & Gibbon, 1995); and at 8, 13 and 16 years via the Kiddie Schedule for Affective Disorders and Schizophrenia, Present and Lifetime Version (Kaufman et al., 1997). These measures yielded lifetime history of depressive disorder (present/absent). At 22 years participants completed the Centre for Epidemiological Studies Depression Scale (CESD) (Radloff, 1977), a standard self-report measure of current symptoms.

#### Emotion regulation task

The experimental task was a variant of that used previously to study emotion regulation in healthy (Lee, Heller, van Reekum, Nelson & Davidson, 2012) or clinically depressed populations (Johnstone et al., 2007), which allows normative emotional processing during passive viewing to be compared with effortful up- and down-regulation of emotion. The paradigm has established validity: attempts to modulate emotional responding in this context are associated with predictable changes in objective measures of emotion (corrugator electromyogram; cEMG) (Jackson et al., 2000); individual differences in neural responding and behaviour (cEMG) are correlated (Lee et al., 2012); and stability in individual responding has been demonstrated (Lee et al., 2012). Participants were scanned while viewing a sequence of 60 emotionally positive and 60 negative pictures taken from the International Affective Picture System (IAPS) (Lang, Bradley, & Cuthbert, 1999). Positively and negatively valent pictures differed on the pleasant/unpleasant dimension (*M *=* *7.58, *SD *= 0.32, and *M *=* *2.39, *SD *= 0.48, respectively, where 1 = most unpleasant and 9 = most pleasant), but were matched on other key dimensions (arousal, sociality, complexity).^1^ In each trial, a 1 s duration cross coupled with a tone was followed by presentation of the IAPS picture for 10 s, and finally by a 6 s blank screen. At the onset of each picture, participants judged the image to be positive or negative via a button press. Four seconds into each presentation, an audio prompt instructed the participant to either ‘increase’ or ‘decrease’ their emotional response to the picture, or to continue to ‘attend’. All audio commands were of 1 s duration and the same sound intensity. There were 20 repetitions of each regulation condition and 12 repetitions of the attend condition for each picture valence, evenly distributed over six runs of 380 s duration. Stimuli were presented in a pseudo-randomised order using a Nordic Neuro Labs head coil mounted LED goggle system. Prior to being scanned, participants completed a brief training during which they viewed a separate set of IAPS pictures and practised re-evaluating images to modify their emotional responses based on standard, written instructions. Scanning was performed using a 3 Tesla Siemens MAGNETOM Trio MRI scanner with a 12-channel Head Matrix coil. Full details of scanning data acquisition and processing are presented in Appendix S1.

On completion of the scanning task, participants used visual analogue scales to rate their ability to comply with regulation instructions (−50 ‘not at all well’ to +50 ‘extremely well’). They also rated their emotional responses to pictures for each condition (i.e., while attending to and regulating for both positive and negative pictures) on two scales indexing affect (−50 ‘extremely negative/depressed’ to +50 ‘extremely positive/happy’) and arousal (−50 ‘extremely agitated’ to +50 ‘extremely relaxed’), which were averaged for analytic purposes.

### fMRI analysis

A first level General Linear Model (GLM) modelled activation for each run using a linear combination of regressors obtained by convolving the known temporal profile of each of the six experimental conditions (2-negative/positive pictures × 3 regulation conditions- up/down or passive) with the double gamma haemodynamic function of FSL. Time derivatives and six estimated motion covariates were also included. Two contrasts were examined: ‘decrease’ emotion versus ‘attend’ to negative pictures; and ‘increase’ emotion versus ‘attend’ to positive pictures. These contrasts from the single run analyses were aggregated across the six runs in a second level fixed effect GLM analysis for each participant. Resulting contrast maps were normalised to MNI space by applying the transformation parameters estimated using FLIRT. As a quality check we verified that the pattern of neural responding for all participants combined during the emotion regulation task corresponded to that observed in previous studies. For the negative pictures, the ‘decrease’ versus ‘attend’ contrast revealed several prefrontal brain regions with relatively greater activation in the decrease condition. These results replicated earlier findings of PFC involvement in the down-regulation of negative affect, with widespread activation in bilateral PFC and the ACC (Heller et al., 2009; Johnstone et al., 2007). With respect to positive pictures, the ‘increase’ versus ‘attend’ contrast again yielded results consistent with previous studies, in particular showing activation in medial PFC. In sum, the task was performing as expected in terms of the overall pattern of neural activity.^2^

To test hypotheses, contrast images from the second level analysis described above (i.e., capturing relative differences in activation during regulate versus attend conditions, for both positive and negative pictures) were entered into two mixed-effects GLMs: in each case subject was a random factor, and attachment (secure vs. insecure) was the between subjects factor. Correction for multiple comparisons was applied using cluster thresholding and Gaussian Random Field Theory (Worsley, Evans, Marrett & Neelin, 1992) to arrive at a corrected *p* < .05. We restricted the analysis *a priori* to regions of the brain previously shown to be involved in active emotion regulation (Johnstone et al., 2007; Ochsner et al., 2004; Phan et al., 2005), including orbitofrontal cortex, cingulate gyrus, superior, middle and inferior frontal gyri. Gender, history of depressive disorder, current depressive symptoms and maternal PND status^3^ were examined as key potential covariates. ROI analysis was also performed within two specific subcortical brain regions, in accordance with hypotheses; bilateral amygdala and NAcc. Masks were created using the Oxford subcortical brain atlas in FSL. Mean percent BOLD signal change was extracted from amygdala/NAcc for each contrast of interest and the resulting scores were analysed using repeated measures ANOVA with laterality (i.e., left vs. right brain), attachment status and gender as independent variables. Finally, where significant group differences in activation in prefrontal regulatory regions were identified, linear regression probed whether secure versus insecure groups also showed different associations between prefrontal and amygdala/NAcc activity, consistent with previous research in the field.

## Results

### Sample characteristics

Sample characteristics are presented in Table1. There were no significant differences between individuals with secure (*n* = 31) versus insecure (*n* = 23) infant attachment status in terms of mean age, gender, past history of depressive disorder and current depressive symptoms (all *p *>* *.22). However, compared with those who had been securely attached, a higher proportion of participants who had been insecurely attached as infants had mothers with PND, χ^2^(1) = 5.77, *p *=* *.02, as expected based on previous reports with the same sample (Murray, Halligan, Adams, Patterson & Goodyer, 2006). Insecure infant attachments were almost exclusively Type A/avoidant (*n* = 20; 87%).

**Table 1:** Demographic characteristics grouped by secure (*N* = 31) versus insecure (*N* = 23) 18-month attachment status

	Secure	Insecure
Proportion female, *N* (%)	16 (51.6)	8 (34.8)
Age in years, *M* (*SD*)	22.3 (.64)	22.4 (.66)
Social class I, II, II and non-manual, *N* (%)	24 (77.4)	15 (65.2)
Maternal PND, *N* (%)	10 (32.3)	15 (65.2)*
CESD score, *M* (*SD*)	10.3 (10.9)	12.7 (10.5)
Past depression, *N* (%)	8 (25.8)	9 (39.1)

CESD, Centre for Epidemiological Studies Depression Scale.

* *P *<* *.05.

### fMRI analysis of the down-regulation of negative affect

We first examined neural responding to negative pictures in relation to infant attachment status (secure vs. insecure). In voxelwise analyses, the ‘decrease’ versus ‘attend’ contrast did not yield significant effects of attachment status. ROI analysis of bilateral amygdala was conducted for the same contrast using a 2 × 2 × 2 repeated measures ANOVA examining % signal change, with attachment status as a between subjects factor, and condition and laterality as within subjects factors. There was no significant attachment group x condition interaction (*F*_1,52_ = 2.81, *p *=* *.10, partial η^2^ = .05), and no attachment x condition x laterality interaction (*F*_1,52_ = 2.33, *p *=* *.13, partial η^2^ = .04). Thus, there was no evidence that 18-month attachment security predicted neural responding in the active down-regulation of emotional responses to negative stimuli.

### fMRI analysis of the up-regulation of positive affect

**Figure 1:**
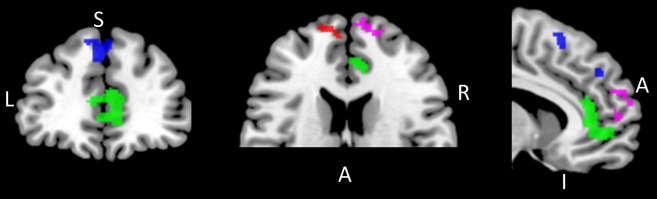
Attachment based differences in the BOLD response to the up-regulate versus passive viewing of positive pictures; indicated areas showed relatively greater activation during the up-regulate condition in the insecure versus secure group. Significant differences were found in the rostral anterior cingulate cortex (green), dorsal medial prefrontal cortex (blue) and the right (pink) and left (red) anterior prefrontal cortex

For the positive stimuli, voxelwise analyses of the ‘increase’ versus ‘attend’ contrast yielded a number of brain regions that were significantly more active in the insecure attachment group than in the secure group. Effects were unchanged when maternal PND (present/absent), current depressive symptoms (CESD), past history of depression (present/absent), and gender were also included in the model. Results, including gender, past depression, CESD scores and maternal PND as covariates, are presented in Table2 and Figure1. Significant differences were identified in four regions; left and right anterior PFC/frontal pole, rostral ACC (rACC), and dorsal medial prefrontal cortex (dmPFC). In each case, greater activation was observed in the insecure than the secure group during up-regulation relative to passive viewing of positive pictures (i.e., increase vs. attend).

**Table 2:** Brain regions significantly more activated in the insecure (*n* = 23) versus secure infant attachment group (*n* = 31) during the ‘increase’ relative to ‘attend’ positive condition. Coordinates locate each cluster's maximum activated voxel in MNI space

Cluster Index	Volume (mm^3^)	X	Y	Z
L anterior PFC/frontal pole (BA 10)	1,384	−22	64	10
R anterior PFC/frontal pole (BA 10)	3,664	38	52	−2
L&R rostral ACC (BA 24)	3,184	2	44	12
L&R dorsal medial PFC (BA 8/9)	2,680	2	42	46

L, left; R, right; BA, Brodmann area; PFC, prefrontal cortex; ACC, anterior cingulate cortex.

**Figure 2:**
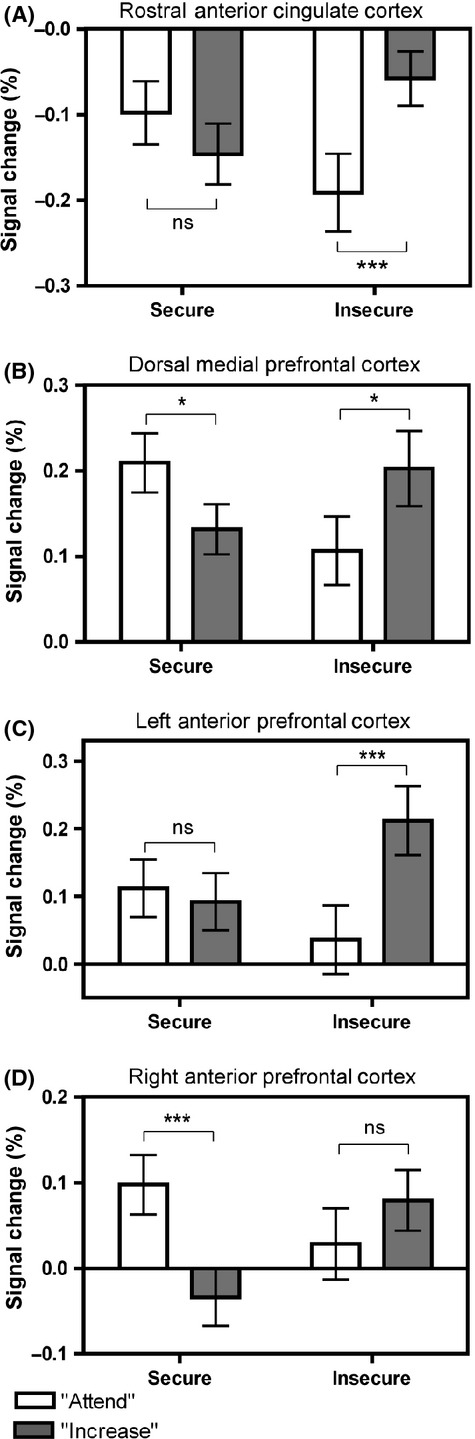
Summary of attachment based differences (secure vs. insecure) in activation in response to passive (‘attend’) versus up-regulate (‘increase’) conditions during the viewing of positive pictures; *p <* *.05, ***p <* *.005

In post hoc analyses which probed these interactions between attachment status and condition, extracted contrast estimates from each cluster were analysed, using paired samples *t-*tests to compare % signal change response between the increase and attend conditions separately for each attachment group. Results are shown in Figure2. In each region, the secure group showed greater activation during the passive viewing of positive pictures compared with the up-regulation condition; this difference (increase < attend) was statistically significant for the dmPFC and right anterior PFC/frontal pole, but not for the rACC or left anterior PFC/frontal pole. By contrast, the insecure group showed significantly greater activation during the up-regulation condition than during passive viewing (increase > attend) in the rACC, left anterior PFC/frontal pole and dmPFC, but not for the right anterior PFC/frontal pole.^4^

We conducted further analyses to investigate whether intervening maternal behaviour at 5 years and 8 years could account for the observed infant attachment effects. This was not the case: multivariate analysis of variance indicated that attachment group effects were a significant predictor of extracted contrast estimates (% signal change for increase vs. attend) even when scores from the two parenting observations were included as covariates (Pillai's trace *F*_4,41_ *= *3.42, *p *=* *.017; partial η^2^=0.25). Follow-up univariate tests confirmed that attachment effects remained significant for each cluster (all *p *<* *.014).

Next, we conducted ROI analysis of NAcc responding; 2 × 2 × 2 repeated measures ANOVA examining NAcc % signal change, with attachment status, condition and laterality as predictors. We found no significant attachment × condition (*F*_1,52_ = 2.81, *p *=* *.10, partial η^2^ = .05), or attachment x condition x laterality (*F*_1,52_ = 2.33, *p *=* *.13, partial η^2^ = .04) interactions. Potential covariates (gender, maternal PND, depression history and current symptoms) were unrelated to NAcc activation and did not alter these null findings when included in the model.

Finally, as the rACC and dmPFC have previously been identified as components of the emotion regulatory circuitry that exert influence over the NAcc, further analyses probed whether links between their activation and NAcc responding differed by attachment group. First, regression analysis examined the prediction of associations between regulatory activation (i.e., increase minus attend) in NAcc by regulatory activation in rACC, attachment status and the rACC activation by attachment status interaction (*R*^2^* *=* *.38, *F *=* *10.1, *df* = 3,50*, p < *.001). Results indicated a significant main effect of attachment status (std. β* = *−.32, *p *=* *.012; coding 0 = secure, 1 = insecure) and a significant interaction term (β* = *−.34, *p *=* *.049). Post hoc regression analyses used to probe the attachment status by rACC activation interaction (Holmbeck, 2002) demonstrated that there was a positive association between rACC and NAcc activation which was stronger for the secure (β = .87, *p *<* *.001) versus insecure (β = .38, *p = *.036) group. Second, an equivalent analysis focusing on dmPFC activation (*R*^2^* *=* *.28, *F *=* *6.34, *df* = 3,50*, p = *.001) identified both dmPFC (β *= *.60, *p *=* *.002) and attachment status (β* = *−.34, *p *=* *.015) to be significant predictors of NAcc activation, but the interaction term was not significant in this case (β* = *−.03, *p *=* *.88).

### Behavioural data

Attachment status effects were explored further through analysis of behavioural data (see Table3). First, we confirmed that secure and insecure groups correctly identified picture valence at similar rates (positive *F*_1,50_ = 0.37, *p *=* *.55; negative *F*_1,50_ = 0.56, *p *=* *.46). Second, we found no significant attachment effects in relation to self-rated ability to comply with task regulatory instructions (increase positive *F*_1,50_ = 1.31, *p *=* *.26; decrease negative *F*_1,50_ = 0.13, *p *=* *.72). Third, we compared self-rated emotional responses during passive viewing of positive pictures to those for the increase condition. Repeated measures ANOVA – with self-rated emotion in the two conditions as within subjects factor, and attachment status and gender as between subjects factors – indicated a trend for a condition by attachment status interaction (*F*_1,50_ = 3.68, *p *=* *.061). Secure and insecure participants reported similar emotional responses while attending to the pictures, but secure participants reported greater positive emotion in the increase condition. Finally, we investigated whether any correspondence existed between reported change in positive emotion (i.e., regulate minus attend), and equivalent neural activation in rACC/dmPFC/antPFC for each attachment group. Separate regression analyses were conducted for each brain region, with change in self-rated emotion as the dependent variable; and gender, neural activation, attachment status, and the neural activation by attachment interaction as predictors. Results indicated that a significant proportion of variance was explained only for the models examining dmPFC (*R*^2^* *=* *.19, *F *=* *2.86, *df* = 4,48*, p *=* *.033) and left anterior PFC activation (*R*^2^* *=* *.27, *F *=* *4.29, *df* = 4,48*, p *=* *.004). For the left anterior PFC, regression coefficients indicated a significant main effect of attachment group (β = −.45, *p *=* *.003), and an attachment status by activation interaction (β = .49, *p *=* *.013) in predicting change in positive emotion. Post hoc probing identified a significant association between left anterior PFC activation and increase in positive emotion for the insecure group (β = .69, *p *=* *.003), but not the secure group (β = .04, *p *=* *.83). For the dmPFC, again the attachment status by activation interaction term was significant (β = .47, *p *=* *.016). In this case, a positive association was observed between activation and change in emotion for the insecure group (β = .42, *p *=* *.061) and a negative association for the secure group (β = −.34, *p *=* *.093), although neither association was statistically significant.

**Table 3:** Behavioural responses to the emotion regulation task, reported by secure (*N* = 31) versus insecure (*N* = 23) 18-month attachment status; means (standard deviations)

	Secure	Insecure
Positive pictures^a^		
% correct valence judgment	96.4 (3.5)	97.5 (2.8)
Compliance with increase instruction^b^	23.9 (20.1)	18.4 (24.1)
Attend condition emotion rating	17.6 (21.3)	15.9 (15.9)
Increase condition emotion rating	31.0 (32.3)	20.7 (26.2)
Negative pictures^a^		
% correct valence judgement	91.7 (6.1)	92.8 (3.5)
Compliance with decrease instruction^b^	11.0 (20.1)	12.7 (21.9)
Attend condition emotion rating	−22.4 (27.6)	−12.3 (22.7)
Decrease condition emotion rating	−9.7 (26.5)	−10.3 (20.2)

a Except for categorical valence judgements, scores ranged from −50 to +50, with higher scores indicating better compliance/more positive emotions.

b Self-reported ability to follow instructions.

## Discussion

In a unique prospective longitudinal study, we demonstrated significant associations between infant attachment security, as measured objectively in a lab-based assessment at 18-months of age, and neural responding to emotional stimuli at 22-years of age. Our observations support the conclusion that the neural underpinnings of emotion regulation may be directly shaped by the quality of the early environment (Burghy et al., 2012), and they also provide support for a key assumption of attachment theory, that the neurobiological systems that subserve adaptive emotional responding are fundamentally linked to the organisation of the early infant-caregiver relationship.

The early attachment relationship is held to be a psychobiological system within which emotional regulation develops and becomes established. In the current study, we demonstrated that young adults who had been insecurely attached infants at 18-months of age showed different neural responding from their securely attached counterparts when actively trying to up-regulate their experience of positive affect relative to a passive viewing baseline. Specifically, when young adults were required to enhance *positive* emotion, those with a history of early insecure infant-mother attachment showed relatively greater activation in the rACC, dmPFC and bilateral anterior PFC/frontal pole than those with a secure early attachment. These observations are remarkable, given the 20-year gap between the measurement of attachment status and adult neural functioning.

The rACC and dmPFC, which form part of the prefrontal top-down emotion regulation circuitry, have particularly dense connections to the striatum, a subcortical region central to processing reward and pleasure-related stimuli. The rACC in particular has extensive projections to the NAcc (Haber, Kim, Mailly & Calzavara, 2006), and functional connectivity between these regions has been established, which is partially underpinned by dopaminergic activity (Nagano-Saito et al., 2008). In the current study, post hoc analyses indicated that rACC engagement during the regulation of positive emotion was less strongly correlated with NAcc activation in insecure versus secure individuals, suggesting that the increased engagement of this prefrontal regulatory region was not associated with a correspondingly greater NAcc response. The medial PFC, including the dmPFC, interfaces between dorsolateral PFC regions involved in cognitive control and reappraisal, and orbitofrontal cortex and limbic regions involved in determining affective responses (Ladouceur et al., 2008). Therefore, overall, the existing literature suggests that greater engagement of the rACC/dmPFC when attempting to increase positive emotion likely reflects a greater deployment of effortful, top-down control in the insecure group. However, other interpretations are also possible. Notably, rACC engagement and recruitment of the dmPFC have been linked with attempts to resolve conflict (Etkin, Egner, Peraza, Kandel & Hirsch, 2006), consistent with the assumption that the insecure group found the effortful reappraisal of positive pictures more challenging. Anterior PFC activation was also observed in the insecure attachment group. This finding is less easily interpreted in an emotion regulation context, but previous research has indicated that activation in this region is indicative of higher level cognitive control, including the coordination of multiple cognitive functions (Ramnani & Owen, 2004). In this regard, it is interesting to note that where participants in the insecure group did show greater anterior PFC activation, this was strongly associated with more effective up-regulation of self-reported positive affect. Thus, the pattern of activation observed again suggests that more extensive cognitive control is being utilised to achieve effective regulation of positive emotion in the insecure group. Finally, we note that in the sample as a whole the emotion regulation task resulted in neural activation in areas previously identified as being central to the active regulation of affect; and there were no PFC regions which the secure group activated more than the insecure group.

Importantly, according to their own report, participants who were insecurely attached in infancy tended to be less effective in up-regulating their positive emotions during our task than their secure counterparts. These behavioural and neural observations are particularly striking in the light of the pattern of insecure attachments in the current sample, which were almost exclusively insecure-avoidant. While ambivalent or disorganised styles may be clearly associated with a failure to contain negative responding, avoidant attachments are held to be characterised by an over-control of negative emotional responses (Weinfield, Sroufe, Egeland & Carlson, 2008). Indeed, research suggests that adults with avoidant attachment styles are adept at containing negative emotions in response to relatively mild stressors (Shaver & Mikulincer, 2007), but they may be less able to experience positive affect, especially in response to social reward (Troisi, Alcini, Coviello, Croce Nanni & Siracusano, 2010). The predominantly avoidant attachments in our group may thus explain the apparently effective regulation we observed in the negative condition (where no attachment effects were demonstrated) but altered neural responding when attempting to up-regulate emotions in the positive context.

No previous study has examined the long-term neural outcomes associated with infant attachment. However, a small number of existing studies have examined concurrent or short-term neural correlates of attachment insecurity in infants and children (Dawson et al., 2001; Gillath, Bunge, Shaver, Wendelken & Mikulincer, 2005), in youth (White et al., 2012), or in relation to adult attachment styles (Strathearn, Fonagy, Amico & Montague, 2009; Vrticka, Andersson, Grandjean, Sander & Vuilleumier, 2008; Zilber, Goldstein & Mikulincer, 2007), albeit without directly targeting emotion regulation processes. These studies also point to altered neural responding in the domain of positive affect, particularly when the focus is on avoidant attachment traits. Thus, reductions in event related potentials linked to approach/reward have been demonstrated in youth with avoidant/dismissing attachments (White et al., 2012); and reduced striatal responding to rewarding stimuli has been found in fMRI studies of adults with avoidant attachment traits (Vrticka et al., 2008), and of mothers with an avoidant attachment style (Strathearn et al., 2009). By contrast, anxious adult attachment traits, which are characterised by ‘hyperactivation’ of the attachment system and correspondingly high emotionality and sensitivity to signs of acceptance/rejection, have been linked particularly to neural responding to negative stimuli, including: greater activation of emotion related areas of the brain (Gillath et al., 2005; Vrticka et al., 2008), and reduced activity in emotion regulatory regions (Gillath et al., 2005) in fMRI analysis; and ERP responses consistent with over-commitment of attentional resources to negative stimuli (Zilber et al., 2007). Our study is the first to produce direct evidence that the security of mother–child attachment in the first years of life may exert long-term effects on neural functioning into adulthood.

While our findings are notable, further research is required before strong conclusions concerning mechanisms of effects can be drawn. In particular, although we identified significant differences in the way in which neural systems regulate emotional processing between the insecure and securely attached groups, the interpretation of our findings draws on the pre-existing neuroscience literature and requires confirmation. We found only partial support for links between behavioural indicators of emotional processing and either attachment security or associated aspects of neural activity. Moreover, the relationship we saw between rACC and NAcc activation cannot be assumed to reflect directional influences of the former on the latter. Future research, including comprehensive measures of the experiences and regulation of positive affect, will be important for understanding the connections between the neural processes we have observed and adults' real-world emotion-regulation capacities and broader socio-emotional functioning.

Our study has important strengths, most notably the longitudinal design which includes an early attachment observation, and the well-characterised sample. At the same time, our analyses were based on secure versus insecure attachment status, whereas an examination of each insecure subtype would ideally be conducted. In addition, while our observations are consistent with the notion that early attachment causally influences later brain development, the study is essentially correlational in design and conclusions are thereby limited. Although we found no evidence that aspects of the later mother–child relationship explained our findings, in the absence of strictly comparable measures of the parent–child relationship throughout development, caution is needed in drawing conclusions about whether aspects of the *early* parenting environment are particularly important. It is also possible that underlying individual differences may have contributed to both the emotional response of the infant towards their mother and later neural responding to emotional stimuli in adulthood, particularly considering the generally increasingly role of genetics in children's socioemotional development over time (Fearon, Shmueli-Goetz, Viding, Fonagy & Plomin, 2014). Nevertheless, it is notable that twin studies have convincingly shown that attachment in early life represents a facet of shared environment and not a child's genes (Bokhorst et al., 2003). Regardless of the possible underlying causes, our observation that insecure attachment during infancy may have a measurable impact on the neural circuitry of emotion regulation more than 20 years later highlights the importance of longitudinal studies in understanding the long-term consequences for neural development of early life experiences (Burghy et al., 2012).

## Footnotes

1 Ratings were as follows for positive and negative pictures, respectively: arousal *M *=* *5.3, *SD *= 0.7 and *M *=* *5.5, *SD *= 0.8; sociality *M *=* *0.68, *SD *= 0.47 and *M *=* *0.75, *SD *= 0.44; complexity JPGs *M *=* *125,424, *SD *= 30,514 and *M *=* *114,965, *SD *= 36,892.

2 Data are available on request from SLH.

3 PND group status was a defining feature of the sample and was therefore controlled for in neural analyses, although no PND group effects emerged.

4 Given that maternal PND status was a key defining feature of the sample, in addition to co-varying for PND in our main analyses, we conducted post hoc ANOVAs to confirm that reported effects in the same direction were present when analyses were confined to the control group. A substantial reduction in power resulted from excluding PND group participants (resultant *n* = 29; 21 secure and 8 insecure participants), and two of the originally reported effects were no long statistically significant. However, all reported attachment effects were in the same direction as already described when examined only in the control group.

## Supporting Information

Appendix S1 fMRI data acquisition and processing.
