# Supplementary material for: Making an effort to feel positive: insecure attachment in infancy predicts the neural underpinnings of emotion regulation in adulthood
Source: J Child Psychol Psychiatry. 2014 Jan 8;55(9):999–1008. doi: 10.1111/jcpp.12198 (PMC4263234; doi:10.1111/jcpp.12198)
Supplement: Appendix S1 — fMRI data acquisition and processing. [file jcpp0055-0999-SD1.docx]

***Supplementary appendix for: Making an effort to feel positive: insecure attachment in infancy predicts the neural underpinnings of emotion regulation in adulthood, by Moutsiana* *et al***

**Appendix S1: fMRI Data Acquisition and Processing**

Functional images were acquired using a T2*-weighted gradient-echo, echo planar imaging pulse sequence [30 interleaved transverse slices, phase encoding P to A, 4 mm thickness, 1 mm interslice gap; 64*64 matrix; 192 mm field of view (FOV); repetition time (TR): 2000ms, echo time (TE): 30ms, Flip Angle: 90°; 190 whole-brain volumes per run]. We also collected a B0 image of the magnetic field (30 slices, TR/TE= 488ms/4.92ms) and a high resolution T1-weighted structural image [MP RAGE, 176 x 1mm slices, 1mm isotropicvoxels TE:2.52ms, TR:2020ms, TI:1100ms, FOV:250mm, Flip Angle: 9°).

The data were processed using FEAT (FMRI Expert Analysis Tool) Version 5.98, part of FMRIB's Software Library (FSL: [www.fmrib.ox.ac.uk/fsl](http://www.fmrib.ox.ac.uk/fsl)). We performed fieldmap-based EPI unwarping using PRELUDE+FUGUE, followed by motion correction with MCFLIRT. Functional data were registered to the Montreal Neurological Institute (MNI) template brain using a 2-stage (6 DOF EPI to T1 and 12 DOF T1 to MNI) linear registration. Functional data were highpass filtered (60s cut off) and smoothed using a 5mm full width at half maximum Gaussian kernel. AR(1) correction for serial correlations was applied
